# Preoperative neutrophil-to-lymphocyte ratio as an early risk-stratification and prognostic biomarker in Wilms tumor: a retrospective study

**DOI:** 10.3389/fonc.2026.1807575

**Published:** 2026-05-07

**Authors:** Dong Feng, Chenglong Zhang, Jiangqun Li, Linying Chen, Can Qi

**Affiliations:** 1 Department of Urology, Hengshui Traditional Chinese Medicine Hospital, Hengshui, Hebei, China; 2 Department of Pediatric Surgery, Hebei Children’s Health and Disease Clinical Medical Research Center, Hebei Children’s Hospital, Shijiazhuang, Hebei, China; 3 Department of Clinical Nutrition, Harrison International Peace Hospital, Hengshui, Hebei, China

**Keywords:** biomarker, neutrophil-to-lymphocyte ratio, prognosis, tumor, Wilms tumor

## Abstract

**Objective:**

To evaluate the predictive value of the preoperative neutrophil-to-lymphocyte ratio (NLR) for prognosis in patients with Wilms tumor (WT).

**Methods:**

A retrospective study was conducted involving 178 children with WTwho underwent surgery. The preoperative NLR was calculated from routine blood tests. Receiver operating characteristic (ROC) curve analysis was used to assess the predictive performance of NLR for overall survival (OS) and event-free survival (EFS). The association between NLR and survival outcomes was analyzed using the Kaplan-Meier curves and Cox regression models.

**Results:**

Patients with an elevated pretreatment NLR exhibited more advanced disease and poorer clinicopathological features. ROC analysis indicated high predictive accuracy of NLR for overall survival (OS; AUC = 0.9066) and event-free survival (EFS; AUC = 0.8669). Kaplan–Meier curves showed significantly worse 5-year OS (66.4% vs. 98.6%; P < 0.001) and EFS (56.8% vs. 94.6%; P < 0.001) in the high NLR group, including those with advanced-stage disease. Multivariate analysis confirmed high NLR as an independent adverse prognostic factor for OS (HR = 12.16, P = 0.035) and EFS (HR = 4.00, P = 0.047), after adjustment for tumor stage, metastasis, and pathological type. Bootstrap internal validation confirmed the robustness of the prognostic model (optimism-corrected C-index = 0.90).

**Conclusion:**

Preoperative NLR serves as an independent predictor of prognosis in WTand may represent a convenient and effective biomarker for risk stratification and treatment decision-making.

## 1 Introduction

Wilms tumor (WT), or nephroblastoma, is the most common renal malignancy in children, accounting for approximately 80%–90% of pediatric renal tumors and 5%–7% of all childhood malignancies (1, 2). The peak incidence occurs in children under 5 years of age, with an estimated annual incidence of 8–9 cases per million in North America and Europe, and approximately 4.3 cases per million in East Asia (1). Over the past five decades, multimodal therapy integrating surgery, chemotherapy, and radiotherapy has raised overall survival (OS) rates to approximately 90% in high-income countries (2). Current treatment follows two principal protocols: upfront nephrectomy with risk-adapted adjuvant chemotherapy (COG) versus preoperative chemotherapy followed by surgery (SIOP) (3, 4). Current risk stratification relies predominantly on postoperative histopathological and molecular markers, including tumor stage, histological subtype, and loss of heterozygosity at chromosomes 1p and 16q (5). However, the postoperative nature of these criteria limits their utility in guiding initial treatment decisions, underscoring the need for readily accessible pretreatment biomarkers.

Systemic inflammation is a recognized hallmark of cancer, contributing to tumor progression and metastasis through cytokine secretion, angiogenesis promotion, and immune suppression (6). In this context, simple hematological indices—most notably the neutrophil-to-lymphocyte ratio (NLR), platelet-to-lymphocyte ratio (PLR), and lymphocyte-to-monocyte ratio (LMR)—have emerged as prognostic biomarkers in pediatric solid tumors (7, 8).

In Wilms tumor specifically, accumulating evidence supports the prognostic utility of these inflammatory markers. Kunc et al. systematically evaluated NLR, PLR, and LMR in 88 pediatric WT patients, demonstrating that elevated preoperative NLR was significantly associated with higher tumor stage and shorter relapse-free survival (9). A larger multicenter study of 235 children by Acker et al. confirmed that elevated NLR, PLR, and LMR at diagnosis were independently associated with higher disease stage, cancer recurrence, and unfavorable histology (10). More recently, Cui et al. identified NLR as an independent prognostic factor for event-free survival in 182 WT patients (HR = 0.40, P = 0.027) (11). Beyond NLR and PLR, other composite indices such as the systemic immune-inflammation index (SII) and pan-immune-inflammation value (PIV) have also shown promise in pediatric solid tumors, though data in WT remain limited (9, 11).

Despite these encouraging findings, the prognostic value of NLR in WT remains incompletely defined, with no consensus regarding optimal cutoff values and limited validation in Asian pediatric populations. Therefore, this study aims to systematically evaluate the association between preoperative NLR and survival outcomes in a Chinese cohort of children with WT, seeking to validate its potential as a readily accessible biomarker for early risk stratification.

## 2 Methods and study population

### 2.1 Patient selection

This retrospective study analyzed the clinical data of patients with WT who underwent surgical treatment at our institution between January 2013 and January 2025. The inclusion criteria were: (1) pathologically confirmed WT, (2) completion of radical tumor resection, and (3) availability of complete preoperative blood test results and follow-up data. The exclusion criteria included severe infection or immunodeficiency, other malignant tumors, and incomplete serological or follow-up data. A total of 178 patients were ultimately enrolled. Based on histological characteristics, WT were classified as favorable histology (FH) or unfavorable histology (uFH), the latter encompassing focal anaplastic and diffuse anaplastic types.

### 2.2 Postoperative adjuvant therapy

Treatment protocols were established in accordance with the CCCG-WT-2009, CCCG-WT-2015, and CCCG-WT-2019 guidelines issued by the Chinese Children’s Cancer Group (CCCG). Patients were stratified into early-stage (Stages I-II) and advanced-stage (Stages III-IV) groups. All patients received standard postoperative chemotherapy. The early-stage group was treated with the EE-4A regimen (vincristine + actinomycin D) for 19 weeks. In contrast, the advanced-stage group received the DD-4A regimen (vincristine + actinomycin D + doxorubicin) for 25 weeks, supplemented by radiotherapy (180 cGy/day, 5 days/week). Drug dosages were administered on day 1 of each cycle. Vincristine was given as an intravenous bolus at 0.025 mg/kg for patients aged <1 year, 0.05 mg/kg for ages 1–3 years, and 1.5 mg/m² (maximum 2.0 mg) for ages >3 years. Actinomycin D was administered as an intravenous infusion at 0.023 mg/kg for ages <1 year and 0.045 mg/kg (maximum 2.3 mg) for ages ≥1 year. For the advanced-stage group, doxorubicin was added via intravenous infusion at 1 mg/kg for patients aged ≤1 year and 30 mg/m² for those >1 year.

### 2.3 Data collection

Clinical, pathological, and laboratory data were collected retrospectively. To exclude the potential influence of neoadjuvant chemotherapy, complete blood count parameters were obtained from all patients under fasting conditions within one week prior to surgery. At the time of blood draw, all patients were afebrile (axillary temperature <37.3 C) and showed no signs of active infection or chronic inflammation. The measured parameters included neutrophil and lymphocyte counts. The NLR was calculated as the absolute neutrophil count divided by the absolute lymphocyte count. Informed consent was obtained from all participants, and the study protocol was approved by the Medical Research Ethics Committee of Hebei Children’s Hospital (202407-90).

### 2.4 Follow-up protocol

All patients were followed up every 3 months for the first 3 years postoperatively, and every 6 months thereafter. To ensure adequate observation time for meaningful survival analysis, patients were required to have a minimum postoperative follow-up period of 6 months, unless an outcome event (death or disease progression) occurred earlier. Patients who did not meet this minimum follow-up requirement without experiencing an event were excluded. During follow-up visits, clinical history was taken, physical examinations were performed, peripheral tumor marker levels were assessed, and abdominal CT scans or ultrasonography were conducted in accordance with the NCCN Clinical Practice Guidelines in Oncology. Overall survival (OS) was defined as the time from the initiation of treatment to death from any cause. Event-free survival (EFS) was defined as the time from the initiation of treatment to the first occurrence of disease progression or recurrence.

### 2.5 Statistical analysis

Statistical analyses were performed using R (version 4.2.2) and GraphPad Prism 9.5.1.733. Continuous variables were presented as medians and compared using the Mann-Whitney U test. Categorical variables were presented as numbers and compared using the Chi-square test. Patients were dichotomized into low-NLR and high-NLR groups based on the median value of the preoperative NLR in the entire study cohort. This approach was chosen to ensure objective and unbiased stratification with approximately equal group sizes for statistical comparison. The predictive efficacy of the NLR for prognosis was assessed by receiver operating characteristic curve analysis. Survival curves were plotted using the Kaplan-Meier method and compared with the log-rank test. Univariate and multivariate Cox proportional hazards regression models were employed to identify independent prognostic factors for OS and EFS. A two-sided P-value < 0.05 was considered statistically significant. To assess the robustness of the prognostic model and correct for potential overfitting, internal validation was performed using bootstrap resampling with 1000 iterations. Model performance was evaluated using Somers’ Dxy and the concordance index (C-index), with optimism-corrected estimates and 95% confidence intervals reported.

## 3 Results

### 3.1 Clinical baseline data

The median preoperative NLR in the entire cohort was 1.04 (0.10–20.83). Based on this median value, patients were categorized into a low-NLR group (NLR ≤ median, n=88) and a high-NLR group (NLR > median, n=90). Compared with Low NLR group, patients in High NLR group were significantly older (median age, 48.0 months vs 12.0 months; p<0.001) and had higher mortality (24.4% vs 1.1%; p<0.001). High NLR group also had a higher prevalence of chemotherapy (85.6% vs 55.7%; p<0.001), distant metastasis (26.7% vs 4.5%; p<0.001), and significant differences in clinical stage distribution (p<0.001), tumor diameter (p<0.001), and pathological type (p=0.038). Sex distribution was similar between groups (p=0.233) (**Table 1**). Absolute neutrophil and lymphocyte counts stratified by age group are presented in **Table 2**. Consistent with normal pediatric immune development, ANC increased progressively with age while ALC declined, resulting in an age-dependent increase in NLR.

**Table 1:** Basic characteristics and differential analysis.

Variables	Total(n = 178)	Low NLR group (n = 88)	High NLR group (n = 90)	Statistic	*P*
Age, M (Q₁, Q₃)	32.00 (12.00, 60.00)	12.00 (5.00, 41.50)	48.00 (12.50, 72.00)	Z=-5.43	**<.001**
Sex, n(%)				χ²=1.42	0.233
Male	95 (53.37)	43 (48.86)	52 (57.78)		
Female	83 (46.63)	45 (51.14)	38 (42.22)		
TumorDiameter(cm), n(%)				χ²=11.90	**<.001**
<5.7	90 (50.56)	56 (63.64)	34 (37.78)		
≥5.7	88 (49.44)	32 (36.36)	56 (62.22)		
Metastatic, n(%)				χ²=16.43	**<.001**
No	150 (84.27)	84 (95.45)	66 (73.33)		
Yes	28 (15.73)	4 (4.55)	24 (26.67)		
Pathological Type, n(%)				χ²=4.30	**0.038**
FH	114 (64.04)	63 (71.59)	51 (56.67)		
uFH	64 (35.96)	25 (28.41)	39 (43.33)		
Clinical Stages, n(%)				χ²=22.92	**<.001**
I~II	117 (65.73)	73 (82.95)	44 (48.89)		
III~IV	61 (34.27)	15 (17.05)	46 (51.11)		
Chemotherapy, n(%)				χ²=19.20	**<.001**
No	52 (29.21)	39 (44.32)	13 (14.44)		
Yes	126 (70.79)	49 (55.68)	77 (85.56)		
Death, n(%)				χ²=21.48	**<.001**
No	155 (87.08)	87 (98.86)	68 (75.56)		
Yes	23 (12.92)	1 (1.14)	22 (24.44)		

Z, Mann-Whitney U test; χ², Chi-square test; M, median; Q₁, first quartile; Q₃, third quartile; FH, favorable histology; uFH, unfavorable histology; NLR, Neutrophil-to-Lymphocyte Ratio. The bold text indicates P values < 0.05, highlighting statistically significant results.

**Table 2:** Absolute neutrophil and lymphocyte counts stratified by age group.

Age group	n	ANC (×10⁹/L) median (IQR)	ALC (×10⁹/L) median (IQR)	NLR (median ANC/Median ALC)
≤ 1 year	70	2.58 (1.82–3.71)	3.80 (2.67–5.41)	0.68
>1 to ≤3 years	35	2.78 (2.30–3.52)	2.56 (1.63–3.77)	1.09
> 3 years	73	2.97 (2.27–4.00)	2.16 (1.69–2.79)	1.38

ANC, absolute neutrophil count; ALC, absolute lymphocyte count; NLR, neutrophil-to-lymphocyte ratio; IQR, interquartile range.

### 3.2 Predictive efficacy of NLR for prognosis

**Figure 1:**
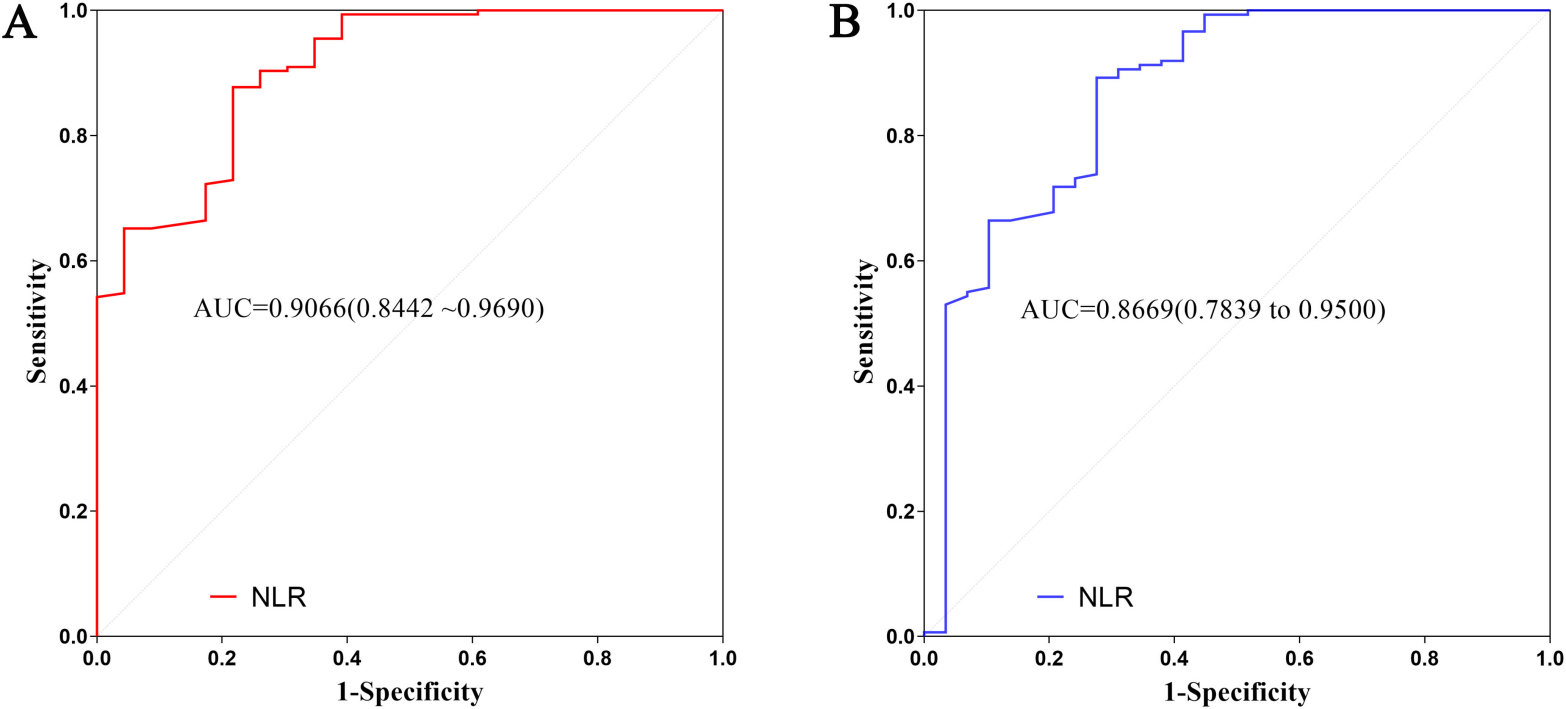
ROC, Receiver operating characteristic curves for the preoperative NLR in predicting survival outcomes in patients with Wilms tumor (n = 178). **(A)** ROC curve for predicting OS, overall survival. The AUC, area under the curve was 0.9066 (95% CI: 0.8442–0.9690). **(B)** ROC curve for predicting EFS, event-free survival. The AUC was 0.8669 (95% CI: 0.7839–0.9500).

To evaluate the prognostic value of the NLR in patients with WT, we constructed ROC curves and calculated the area under the curve (AUC) using death and disease progression as endpoints. As shown in **Figure 1A**, the ROC analysis for death demonstrated significant predictive efficacy for NLR, with an AUC of 0.9066 (95% CI: 0.8442–0.9690). In the analysis for disease progression (**Figure 1B**), NLR also exhibited strong predictive performance, yielding an AUC of 0.8669 (95% CI: 0.7839–0.9500).

### 3.3 Stratification analysis based on NLR and survival outcomes

**Figure 2:**
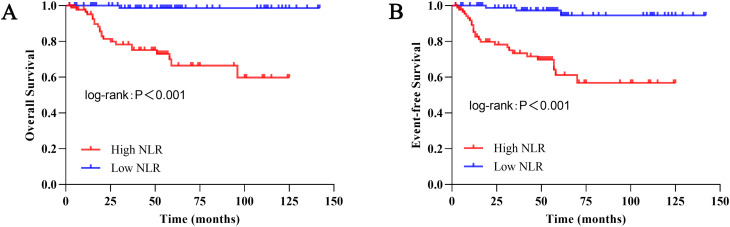
Kaplan–Meier survival curves for patients with Wilms tumor stratified by preoperative NLR (n = 178). Patients were dichotomized into low-NLR (n = 88) and high-NLR (n = 90) groups based on the median preoperative NLR value of the entire cohort. **(A)** OS, Overall survival. The 5-year OS rate was 98.6% in the low-NLR group and 66.4% in the high-NLR group (P < 0.001, log-rank test). **(B)** EFS, Event-free survival. The 5-year EFS rate was 94.6% in the low-NLR group and 56.8% in the high-NLR group (P < 0.001, log-rank test).

Kaplan-Meier survival curves were generated for OS and EFS, and between-group differences were evaluated using the log-rank test. Kaplan-Meier analysis demonstrated that NLR served as a significant prognostic factor. As illustrated in **Figure 2A**, the high NLR group exhibited significantly inferior OS compared to the low NLR group (5-year OS: 66.4% vs. 98.6%; P < 0.001). Similarly, EFS was also markedly lower in the high NLR group (5-year EFS: 56.8% vs. 94.6%; P < 0.001)(**Figure 2B**).

**Figure 3:**
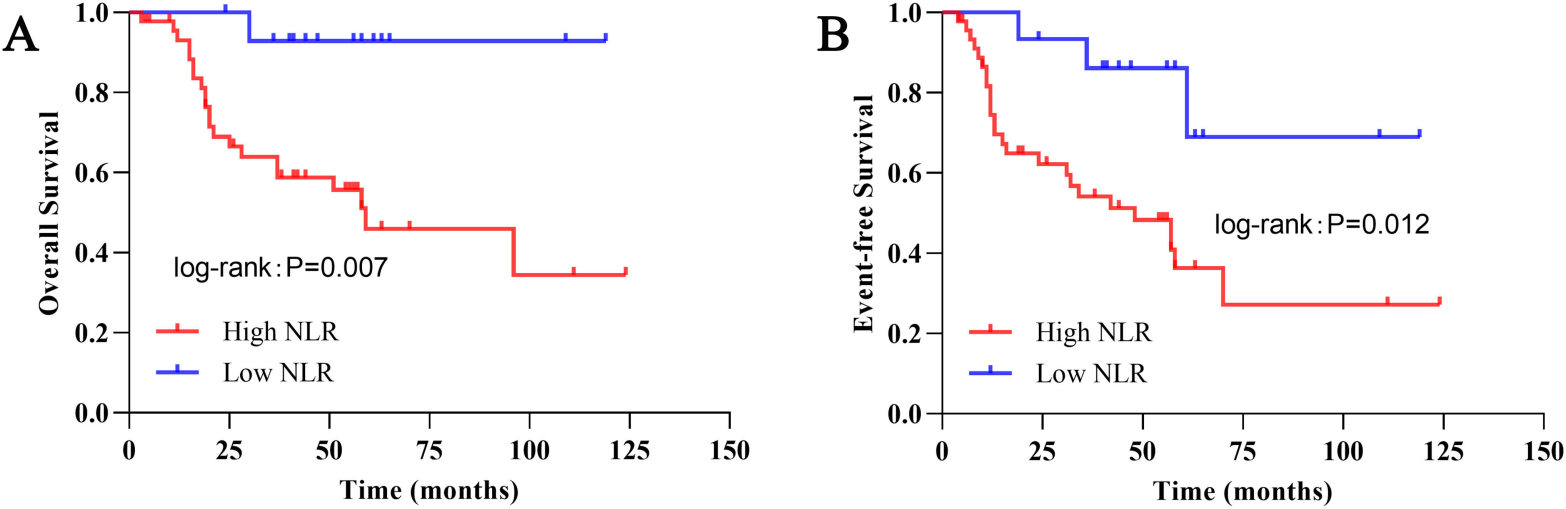
Kaplan–Meier survival curves for patients with advanced-stage (III–IV) Wilms tumor stratified by preoperative NLR (n = 61). Among patients with advanced-stage disease, those were further dichotomized into low-NLR (n = 15) and high-NLR (n = 46) groups based on the median preoperative NLR value of the entire cohort. **(A)** OS, Overall survival. The 5-year OS rate was 92.9% in the low-NLR group and 46.0% in the high-NLR group (P = 0.007, log-rank test). **(B)** EFS, Event-free survival. The 5-year EFS rate was 86.2% in the low-NLR group and 36.3% in the high-NLR group (P = 0.012, log-rank test).

Subgroup analysis restricted to patients with advanced-stage (III–IV) disease further corroborated these findings. Among these patients, those were further stratified into low-NLR (n = 15) and high-NLR (n = 46) groups using the same median cutoff derived from the entire cohort. As shown in **Figure 3**, patients with high NLR continued to exhibit significantly worse OS (5-year OS: 46.0% vs. 92.9%; P = 0.007) (**Figure 3A**) and EFS (5-year EFS: 36.3% vs. 86.2%; P = 0.012) (**Figure 3B**) relative to the low-NLR group.

### 3.4 Cox regression analysis for OS

To determine the independent prognostic value of the NLR for OS in patients with WT, univariate and multivariate Cox proportional hazards regression analyses were performed. Univariate analysis (**Table 3**) identified several variables significantly associated with poorer OS, including larger tumor diameter (≥5.7 cm; HR = 3.15, 95% CI: 1.24–8.00; P = 0.016), presence of distant metastasis (HR = 86.98, 95% CI: 20.16–375.31; P < 0.001), unfavorable histology (uFH; HR = 18.19, 95% CI: 4.26–77.62; P < 0.001), advanced clinical stage (Stage III–IV; HR = 45.90, 95% CI: 6.18–340.82; P < 0.001), receipt of chemotherapy (HR = 9.50, 95% CI: 1.28–70.55; P = 0.028), and high NLR (HR = 28.65, 95% CI: 3.86–212.73; P = 0.001).

**Table 3:** Univariate and multivariate Cox regression analysis of NLR on OS.

Variables	Univariate		Multivariate	
	HR (95%CI)	*P*	HR (95%CI)	*P*
Age, M (Q₁, Q₃)	1.00 (0.99 ~ 1.01)	0.718	0.99 (0.97 ~ 1.00)	0.132
Sex, n(%)				
Male	1.00 (Reference)		1.00 (Reference)	
Female	0.84 (0.37 ~ 1.91)	0.677	2.10 (0.84 ~ 5.26)	0.113
TumorDiameter(cm), n(%)				
<5.7	1.00 (Reference)		1.00 (Reference)	
≥5.7	3.15 (1.24 ~ 8.00)	0.016	1.40 (0.50 ~ 3.96)	0.523
Metastatic, n(%)				
No	1.00 (Reference)		1.00 (Reference)	
Yes	86.98 (20.16 ~ 375.31)	<.001	39.49 (4.57 ~ 341.46)	**<.001**
Pathological Type, n(%)				
FH	1.00 (Reference)		1.00 (Reference)	
uFH	18.19 (4.26 ~ 77.62)	<.001	0.85 (0.10 ~ 7.27)	0.879
Clinical Stages, n(%)				
I~II	1.00 (Reference)		1.00 (Reference)	
III~IV	45.90 (6.18 ~ 340.82)	<.001	3.49 (0.22 ~ 54.26)	0.372
Chemotherapy, n(%)				
No	1.00 (Reference)		1.00 (Reference)	
Yes	9.50 (1.28 ~ 70.55)	0.028	0.37 (0.03 ~ 4.88)	0.453
NLR, n(%)				
Low	1.00 (Reference)		1.00 (Reference)	
High	28.65 (3.86 ~ 212.73)	0.001	12.16 (1.19 ~ 124.58)	**0.035**

HR, hazard ratio; CI, confidence interval; FH, favorable histology; uFH, unfavorable histology; NLR, Neutrophil-to-Lymphocyte Ratio. The bold text indicates P values < 0.05, highlighting statistically significant results.

Variables demonstrating statistical significance in the univariate analysis were subsequently incorporated into a multivariate Cox model to further evaluate the independence of NLR. After adjusting for confounding factors, the analysis revealed that distant metastasis (HR = 39.49, 95% CI: 4.57–341.46; P < 0.001) and high NLR (HR = 12.16, 95% CI: 1.19–124.58; P = 0.035) remained independent risk factors for OS (**Table 3**).

### 3.5 Cox regression analysis for EFS

The predictive value of NLR for EFS was similarly assessed using Cox regression models. Univariate analysis (**Table 4**) indicated that larger tumor diameter (≥5.7 cm; HR = 3.04, 95% CI: 1.34–6.86; P = 0.008), distant metastasis (HR = 40.15, 95% CI: 16.01–100.72; P < 0.001), unfavorable histology (uFH; HR = 11.26, 95% CI: 3.91–32.39; P < 0.001), advanced clinical stage (Stage III–IV; HR = 65.58, 95% CI: 8.90–483.27; P < 0.001), receipt of chemotherapy (HR = 12.79, 95% CI: 1.74–94.13; P = 0.012), and high NLR (HR = 11.74, 95% CI: 3.54–38.86; P < 0.001) were all significantly associated with shorter EFS.

**Table 4:** Univariate and multivariate Cox regression analysis of NLR on EF.

Variables	Univariate		Multivariate	
	HR (95%CI)	*P*	HR (95%CI)	*P*
Age, M (Q₁, Q₃)	1.00 (0.99 ~ 1.01)	0.439	1.00 (0.99 ~ 1.01)	0.661
Sex, n(%)				
Male	1.00 (Reference)		1.00 (Reference)	
Female	0.89 (0.43 ~ 1.85)	0.756	1.95 (0.85 ~ 4.46)	0.114
TumorDiameter(cm), n(%)				
<5.7	1.00 (Reference)		1.00 (Reference)	
≥5.7	3.04 (1.34 ~ 6.86)	0.008	1.29 (0.53 ~ 3.16)	0.576
Metastatic, n(%)				
No	1.00 (Reference)		1.00 (Reference)	
Yes	40.15 (16.01 ~ 100.72)	<.001	8.95 (2.49 ~ 32.21)	**<.001**
Pathological Type, n(%)				
FH	1.00 (Reference)		1.00 (Reference)	
uFH	11.26 (3.91 ~ 32.39)	<.001	1.75 (0.43 ~ 7.09)	0.432
Clinical Stages, n(%)				
I~II	1.00 (Reference)		1.00 (Reference)	
III~IV	65.58 (8.90 ~ 483.27)	<.001	12.15 (1.25 ~ 118.04)	0.031
Chemotherapy, n(%)				
No	1.00 (Reference)		1.00 (Reference)	
Yes	12.79 (1.74 ~ 94.13)	0.012	0.79 (0.08 ~ 7.76)	0.841
NLR, n(%)				
Low	1.00 (Reference)		1.00 (Reference)	
High	11.74 (3.54 ~ 38.86)	<.001	4.00 (1.02 ~ 15.68)	**0.047**

Z, Mann-Whitney U test; χ², Chi-square test; M, median; Q₁, first quartile; Q₃, third quartile; FH, favorable histology; uFH, unfavorable histology; NLR, Neutrophil-to-Lymphocyte Ratio. The bold text indicates P values < 0.05, highlighting statistically significant results.

Multivariate analysis (**Table 4**), adjusted for potential confounders, demonstrated that distant metastasis (HR = 8.95, 95% CI: 2.49–32.21; P < 0.001), advanced clinical stage (Stage III–IV; HR = 12.15, 95% CI: 1.25–118.04; P = 0.031), and high NLR (HR = 4.00, 95% CI: 1.02–15.68; P = 0.047) were independent predictors of inferior EFS. This result confirms that a high NLR level is an independent predictive factor for composite events (including recurrence, metastasis, or death) in WT patients, associated with an approximately 4-fold increase in risk.

### 3.6 Internal validation of the prognostic model

Bootstrap internal validation (1000 resamples) was performed to evaluate the robustness of the multivariate Cox model incorporating NLR, age, tumor stage, metastasis, and pathological type. The optimism-corrected concordance index (C-index) was 0.90, indicating excellent discriminatory ability. The corrected Somers’ Dxy was 0.80 (95% CI: 0.64–0.92), and the corrected R² was 0.43 (95% CI: 0.24–0.59), confirming the stability and predictive accuracy of the model.

## 4 Discussion

This retrospective study, analyzing clinical data from 178 children with WT, systematically evaluated the prognostic value of the pre-treatment NLR. Our results demonstrate a significant association between elevated NLR and inferior survival outcomes. In multivariate Cox regression analyses, a high NLR emerged as an independent risk factor for both OS and EFS, suggesting its potential utility for risk stratification in WT.

Systemic inflammatory response is widely recognized as a hallmark of cancer, playing a critical role in tumorigenesis, progression, and metastasis (6). The inflammatory microenvironment facilitates disease aggressiveness by promoting angiogenesis, suppressing immune surveillance, and enhancing tumor cell invasiveness (12). In recent years, systemic inflammatory markers derived from peripheral blood cell counts, such as NLR, platelet-to-lymphocyte ratio (PLR), and systemic immune-inflammation index (SII), have gained attention for their significant prognostic value in various solid tumors due to their accessibility, cost-effectiveness, and reproducibility (8, 13–15). Elevated NLR, in particular, has been consistently linked to poor survival in adult cancers including colorectal, hepatocellular, and gastric carcinomas (16, 17). Our study extends this concept to pediatric WT, broadening the applicability of inflammatory biomarkers.

The NLR serves as a surrogate marker of systemic inflammation, reflecting underlying dysregulation of the tumor immune microenvironment. Within the tumor microenvironment, neutrophils typically adopt an N2 tumor-associated neutrophil (TAN) phenotype. These cells promote tumor angiogenesis, local invasion, and distant metastasis by secreting substances such as matrix metalloproteinases (MMPs), vascular endothelial growth factor (VEGF), and reactive oxygen species (ROS) (18). Furthermore, neutrophils enhance metastatic seeding by releasing neutrophil extracellular traps (NETs), which capture circulating tumor cells (19). In contrast, lymphocytes—particularly cytotoxic T cells and natural killer cells—are key mediators of anti-tumor immunity. Inadequate lymphocytic infiltration or functional impairment can lead to failure of immune surveillance, allowing tumor cells to evade immune elimination (20). Thus, a high NLR (indicating elevated neutrophil counts and reduced lymphocyte counts) essentially reflects an immune state characterized by “pro-tumor dominance and anti-tumor deficiency.” This imbalance has been demonstrated in multiple cancers to correlate with poor prognosis (21).

Beyond the local tumor microenvironment, alterations in circulating neutrophil and lymphocyte counts also reflect broader systemic tumor-host interactions. Tumors can induce systemic immune modulation through the release of soluble factors—such as granulocyte colony-stimulating factor (G-CSF), interleukin-6 (IL-6), and interleukin-8 (IL-8)—into the circulation. These cytokines stimulate emergency granulopoiesis in the bone marrow and mobilize myeloid-derived suppressor cells (MDSCs), leading to peripheral neutrophilia (22, 23). Concurrently, tumor-driven systemic immune suppression—mediated by factors such as transforming growth factor-β (TGF-β) and prostaglandin E₂ (PGE₂)—impairs lymphocyte proliferation and promotes T-cell apoptosis, resulting in relative or absolute lymphopenia (24). Thus, an elevated NLR serves not merely as a surrogate for the local tumor immune microenvironment but as an integrated indicator of systemic inflammatory and immune dysregulation, capturing the net balance of pro-tumor and anti-tumor forces at the whole-organism level.

Our findings align with and extend the growing body of evidence supporting the prognostic value of NLR in Wilms tumor. Consistent with the seminal report by Kunc et al., we observed that elevated preoperative NLR was significantly associated with higher tumor stage and unfavorable histology, and independently predicted inferior survival (9). Similarly, the multicenter study by Acker et al. confirmed that elevated NLR at diagnosis correlated with advanced stage, recurrence, and anaplastic histology in 235 children (10). Our results further corroborate the recent findings of Cui et al., who identified NLR as an independent prognostic factor for EFS in a Chinese cohort of 182 WT patients (11). Notably, the predictive performance of NLR in our study (AUC: 0.91 for OS, 0.87 for EFS) compares favorably with or exceeds that reported in previous investigations, which may reflect differences in cohort characteristics or cutoff methodologies.

Despite this overall consistency, notable differences exist across studies. First, the optimal NLR cutoff has varied considerably—ranging from median splits (as in our study) to ROC-derived thresholds and age-adjusted values (9). This heterogeneity underscores the lack of a standardized threshold and highlights the need for external validation in diverse populations. Second, patient populations differ geographically and ethnically: our cohort and those of Cui et al. (11) comprise East Asian children, whereas Kunc et al. (9) and Acker et al. (10) predominantly enrolled Caucasian patients. Given that baseline NLR values are influenced by genetic and environmental factors, the consistent prognostic signal across populations strengthens the generalizability of NLR as a robust biomarker. Third, our study uniquely included only patients who underwent upfront surgery without neoadjuvant chemotherapy, thereby avoiding potential confounding effects of preoperative treatment on hematological parameters—a strength not uniformly shared by prior investigations.

The lack of independent association between chemotherapy and survival in multivariate analysis likely reflects confounding by indication. In our cohort, chemotherapy assignment was dictated by disease stage per CCCG protocols. Consequently, chemotherapy status is highly collinear with stage and other prognostic covariates. After adjusting for these factors, the independent effect of chemotherapy was naturally attenuated, as its prognostic benefit is largely captured by the clinical variables that determine its use. This does not diminish the established curative role of chemotherapy in Wilms tumor.

Traditionally, WT has been viewed as an embryonal malignancy arising primarily from aberrant differentiation of renal blastemal cells during kidney development, with inflammatory responses receiving limited attention. However, growing evidence suggests that inflammation not only contributes to the formation of the tumor microenvironment but may also actively drive tumor initiation, progression, and metastasis (6, 18). Infiltration of diverse immune cells—including tumor-associated neutrophils (TANs), M2-type tumor-associated macrophages (TAMs), and regulatory T cells (Tregs)—has been detected in WTtissues. These cells secrete pro-inflammatory factors such as IL-6, TNF-α, and VEGF, which promote angiogenesis, suppress cytotoxic T-cell function, and induce epithelial–mesenchymal transition (EMT), thereby establishing a foundation for tumor metastasis (13, 25). Notably, the inflammatory microenvironment may also influence the cellular origin and differentiation state of WT. Evidence indicates that chronic inflammation may disrupt normal renal developmental processes, driving malignant transformation of metanephric mesenchymal cells or impeding the maturation of WT cells (26).

The key significance of our study lies in demonstrating through multivariable analysis that NLR serves as a predictor independent of traditional prognostic factors such as tumor stage, histology, and metastasis status. This implies that NLR provides unique prognostic information even after accounting for these established risk factors. This finding offers a potential solution to the critical issue raised earlier—that current risk stratification systems heavily rely on postoperative parameters.

Integrating the readily available NLR biomarker into existing risk stratification offers considerable clinical potential. Current risk stratification in WT depends largely on postoperative pathology and molecular markers. Although valuable, these indicators are unavailable before treatment initiation, limiting their utility in formulating initial therapeutic strategies (27, 28). Both the International Society of Pediatric Oncology (SIOP) and the Children’s Oncology Group (COG) have developed increasingly complex risk classification systems that are beginning to incorporate molecular markers such as loss of heterozygosity at 1p and 16q (27–29). However, as emphasized by Dome et al., one consequence of such refined stratification is the division of patients into very small subgroups, making statistically powered clinical trials feasible only through large-scale international collaboration (29). In this context, NLR could help identify high-risk patients with potentially worse prognoses even within the same clinical stage or histologic subtype, as early as at diagnosis. This enables more precise adjustment of treatment intensity from the outset: high-risk patients may require intensified therapy or closer surveillance, whereas those with low NLR and other favorable prognostic factors could potentially qualify for future treatment de-escalation strategies aimed at reducing long-term toxicity—aligning with the overarching goal of “reducing toxicity while maintaining efficacy” in WT management (30, 31). Furthermore, given that inflammation represents a potential therapeutic target, patients identified by high NLR might benefit from future anti-inflammatory adjuvant therapies (e.g., non-steroidal anti-inflammatory drugs or more targeted immunomodulators), opening avenues for novel treatment strategies (4, 12, 32).

An important consideration specific to pediatric oncology is the dynamic maturation of the immune system during childhood, which substantially influences hematological parameters including the NLR. In healthy children, lymphocyte counts predominate in infancy (constituting up to 60% of circulating leukocytes), with a gradual increase in neutrophil counts throughout early childhood; the neutrophil-to-lymphocyte ratio typically reaches approximately 1:1 by 4–6 years of age (33). A recent large-scale study of 2,106 healthy Chinese children established age-specific reference intervals for NLR, demonstrating a clear upward trend from infancy (0.10–0.92 for ages 28 days–2 years) to adolescence (0.57–2.30 for ages 6–18 years) (33). These physiological shifts reflect the gradual transition from lymphocyte predominance in early life to neutrophil predominance in later childhood, driven by the expansion and maturation of the adaptive immune compartment. Indeed, absolute lymphocyte counts have been shown to peak around 1 year of age and then progressively decline through adolescence (34).

In our cohort, we observed that older children had higher NLR values, a finding consistent with this established developmental trajectory. Crucially, age was included as a continuous covariate in all multivariate Cox regression models, and high NLR remained an independent adverse prognostic factor for both OS (HR = 28.65, 95% CI: 3.86–212.73; P = 0.001) and EFS (HR = 4.00, 95% CI: 1.02–15.68; P = 0.047) after this adjustment. This indicates that the prognostic value of NLR in Wilms tumor reflects genuine tumor-host interactions that extend beyond normal age-related immune maturation. Nonetheless, future prospective studies in pediatric oncology would benefit from employing age-adjusted NLR z-scores or percentile-based cutoffs derived from healthy pediatric reference populations to further standardize risk assessment across the diverse age spectrum of Wilms tumor patients.

This study has several limitations. First, it was a single-center retrospective analysis with a limited sample size, and the optimal NLR cutoff has not been validated in multicenter cohorts. Additionally, the relatively modest number of outcome events (23 deaths, 28 metastatic cases) in our cohort contributes to reduced precision of certain hazard ratio estimates, as reflected by the wide confidence intervals observed in the univariate analysis. While the statistical significance and direction of the association are clear, the precise magnitude of the effect warrants cautious interpretation and requires validation in larger, multi-institutional prospective studies. Second, the prognosis of WT is significantly influenced by various molecular genetic features, such as 1p/16q LOH and WT1 mutations. However, due to the retrospective nature and long time span of this study, tumor tissue samples were not available for all patients, preventing uniform and comprehensive genomic analysis. Future work should involve prospective multicenter studies with systematic tumor sample collection and integrated genomic profiling. Third, this study spanned 12 years during which CCCG-WT guidelines underwent several revisions. Although core chemotherapy remained consistent, subtle temporal variations in treatment may have introduced confounding. Treatment era was not included as a covariate due to sample size limitations. Future studies should validate our findings with protocol stratification.

In conclusion, our findings indicate that preoperative NLR may serve as an early, non-invasive, and dynamically monitorable prognostic indicator, assisting clinicians in identifying high-risk patients before treatment and informing decisions regarding chemotherapy intensity or timing of surgery.

## Data Availability

The raw data supporting the conclusions of this article will be made available by the authors, without undue reservation.
